# The Epizooty

**Published:** 1877-04-20

**Authors:** 


THE EPIZOOTY has again broke out among the horses in New York and Brooklyn, and this time with increased mortality. It will be remembered that when it occurred two years ago it was not attended with much fatality—that it spread rapidly from New York southward, and prevailed extensively in the Southern and Western States, affecting both man and beast. In the human subject it manifested itself in a form of influenza attended with sore throat, cough, etc., and was experienced in some degree by nearly everybody. It may be looked for again, and in, perhaps, more agravated form. During its former prevalence we found the administration of quinine and the com. syrup of squills, with the solution of the chlorate of potassa as a gargle, to give the best results in treatment.

